# Sentinel Lymph Node Detection Using Laser-Assisted Indocyanine Green Dye Lymphangiography in Patients with Melanoma

**DOI:** 10.1155/2013/904214

**Published:** 2013-12-08

**Authors:** Vikalp Jain, Brett T. Phillips, Nicole Conkling, Colette Pameijer

**Affiliations:** ^1^Department of Surgery, Stony Brook University Medical Center, Stony Brook, NY 11794-8191, USA; ^2^Stony Brook School of Medicine, Stony Brook University Medical Center, Stony Brook, NY 11794, USA

## Abstract

*Introduction*. Sentinel lymph node (SLN) biopsy is a vital component of staging and management of multiple cancers. The current gold standard utilizes technetium 99 (tech99) and a blue dye to detect regional nodes. While the success rate is typically over 90%, these two methods can be inconclusive or inconvenient for both patient and surgeon. We evaluated a new technique using laser-assisted ICG dye lymphangiography to identify SLN. *Methods*. In this retrospective analysis, we identified patients with melanoma who were candidates for SLN biopsy. In addition to tech99 and methylene blue, patients received a dermal injection of indocyanine green (ICG). The infrared signal was detected with the SPY machine (Novadaq), and nodes positive by any method were excised. *Results*. A total of 15 patients were evaluated, with 40 SLNs removed. Four patients were found to have nodal metastases on final pathology. 100% of these 4 nodes were identified by ICG, while only 75% (3/4) were positive for tech99 and/or methylene blue. Furthermore, none of the nodes missed by ICG (4/40) had malignant cells. *Conclusion*. ICG dye lymphangiography is a reasonable alternative for locating SLNs in patients with melanoma. Prospective studies are needed to better ascertain the full functionality of this technique.

## 1. Introduction 

Sentinel lymph node biopsy has become a standard method of staging lymph node basins for multiple cancers, including melanoma. The status of the lymph node provides valuable prognostic information and helps patients and physicians make decisions about further treatment. The current method uses an injection of technetium99 (tech99) in the cancerous area, with imaging an hour later. This is usually done the day prior to surgery but can be done on the same day. Following induction of anesthesia, the patient is injected with blue dye (methylene blue in our institution, MB), again in the cancerous area. The radioactive signal from the sentinel node is localized with a probe, and the blue dye is a visual aid.

With these two methods, sentinel node localization has a reported success rate of 96–99% [1]. Despite this high reported success rate, both tech99 and blue dye have drawbacks. The tech99 often requires an additional visit to the hospital and is a radioactive substance, and the injection is often painful. MB dye stains surrounding tissues, which can obscure tissue planes, and may be toxic to skin grafts. For these reasons we sought an alternative method of sentinel node localization.

Indocyanine green (ICG) is a green dye with near-infrared fluorescent properties. After dermal injection of ICG, real-time lymphangiography can be performed in the operating room using a laser-assisted imaging device (SPY machine, Novadaq Corp., Bonita Springs, FL). Sentinel node dissection can be performed concurrent with imaging. The goal of this study was to investigate the utility of this method of sentinel node localization compared to tech99 and blue dye.

## 2. Methods 

This is an IRB approved retrospective analysis of patients with melanoma who underwent sentinel node biopsy between April 2010 and January 2011. All patients received a dermal injection of tech99 at the tumor site the day prior to surgery. In the operating room, after induction of anesthesia, 1-2 cc of methylene blue dye and 0.9 cc (2.25 mg) of indocyanine green (ICG) were injected around the tumor. The dose of ICG is the lowest reported dose found in the literature [2]. Ten minutes after injection, the SPY machine was used to detect real time lymphatic flow towards the lymph node basin. The nodal basin of interest was determined primarily by the tech99, although the ICG can often be seen through the skin prior to incision. In these preliminary subjects, the surgeon was not blinded to the results of the tech99 scan and did not attempt to localize the nodes with ICG alone.

A skin incision was made in the lymph node basin (neck, axilla, or groin) using a combination of the gamma probe and lymphatic tracking by the SPY machine. The SPY machine can be used to spot check the basin or node, or for real time dissection. The camera of the SPY machine is positioned over the basin, with the image projected onto a computer screen. Similar to laparoscopy, the surgeon can watch the image and operate at the same time. Sentinel lymph nodes were identified in the corresponding nodal basin using these methods. Any nodes that were “hot” by the gamma probe, blue, or intense with ICG were excised and evaluated as sentinel nodes. Each excised node was assessed for the presence of each dye. Patients were managed as per standard of care, with any malignant lymph nodes dictating further evaluation and care.

## 3. Results 

During this time frame one surgeon operated on a total of 92 patients with melanoma, with 38 having a sentinel node biopsy. Based on availability of the SPY machine and ICG dye, a total of 15 patients were evaluated with the SPY machine, with no adverse reactions to MB or ICG. The mean age was 61 years (range: 40–87). Out of the 15 patients, two did not localize by any of the three methods. In one of these patients, incision was made based on the tech99 scan. A single node was detected clinically and excised. The node was negative, but the patient later presented with in-transit disease in the chest and thereafter with nodal recurrence. He now has no evidence of disease after excision of these recurrences, three years from diagnosis. Given his clinical course, his data will be excluded from further analysis, as he likely did not have the true sentinel node biopsied. The second patient had no identifiable nodes, and no incision was made. She is without recurrence and disease-free. In the remaining 13 patients (87%), nodes were identified by at least one method. Real time ICG lymphangiography was successful in mapping 13 of the 15 patients (87%). ICG did not identify any additional nodal basins over the tech99. Methylene blue identified sentinel nodes in 11 patients (73%) and tech99 in 12 patients (80%). The combination of tech99 and MB was successful in 13 of the 15 patients.

Table 1 shows the primary tumor stage and site for each patient and the corresponding nodal basin(s). The ICG+, MB+, and tech99+ columns indicate how many nodes were positive with each of these methods in each patient. A mean of 2.5 sentinel lymph nodes were removed per patient (range 0–6), with 4 nodes positive in 4 patients. The last column highlights which of the three methods identified the malignant sentinel nodes. The average number of nodes, identified per patient was determined by dividing the total nodes identified by that method, by the number of patients in which that method was successful.

Two patients localized to 2 nodal basins and the remaining patients to one basin. ICG identified 93% (37/40) of the sentinel nodes, tech99 78% (31/40), and methylene blue 45% (18/40). Patients had an average of 2.5 nodes identified by ICG versus 2.1 nodes with tech99. The combination of tech99 and MB identified 83% of sentinel nodes (33/40). Four patients had metastatic disease in the lymph nodes, with all four being identified by ICG (Table 2). Tech99 ± MB identified only 3 of the 4 positive nodes (75%). None of the lymph nodes missed by ICG (4/40) were malignant.

**Figure 1:**
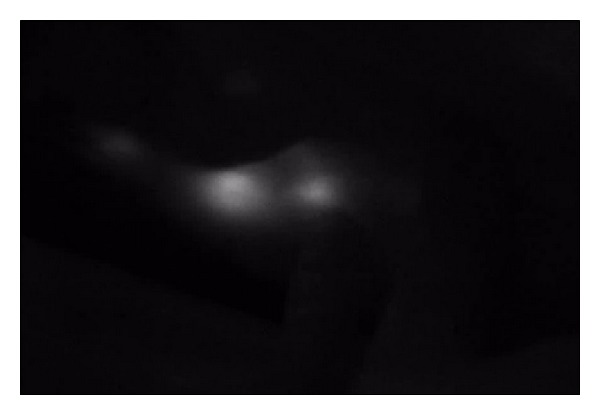
Sentinel nodes imaged with the SPY machine through the skin, prior to incision.

**Figure 2:**
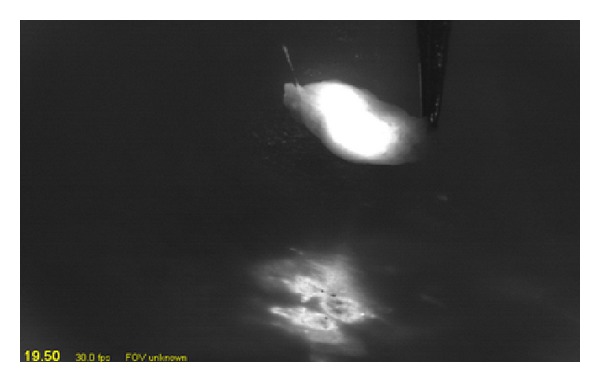
SPY imaging after excision of the sentinel node. The superior object is the SLN. The inferior area is the original chest wall melanoma site.

Real time lymphangiography can expedite identification of sentinel nodes, as the technology allows the surgeon to dissect the basin while visualizing the lymphatics. ICG often fluoresces through the skin, as shown in Figure 1. The sentinel node is typically very bright (Figure 2) and easy to distinguish from surrounding fat or lymphatic vessels. The afferent lymphatic channels are often visualized and can be traced to the sentinel node if the node is not immediately apparent in the field. The SPY machine can quantify the intensity of the fluorescence, although that was not recorded in this study.

## 4. Discussion 

Sentinel lymph node biopsy has become a routine procedure in the staging of melanoma. There is extensive experience with both radiolabeled colloid and blue dyes, with overall a high rate of sentinel node localization [1]. Nevertheless, there are technical drawbacks to each method, and a false negative rate of approximately 10%. This false negative rate seems to increase with age [3, 4]. While the overall survival of patients with a false negative sentinel node is no different than those with a true positive one, there is naturally a higher incidence of local or in-transit recurrence [4]. A false negative can arise for several reasons, including unsuccessful localization. Any method that may improve localization would be of benefit to patient care.

Real time lymphangiography with ICG can also simplify the localization process for patients. The radioactive colloid injection is often done the day prior to surgery, can be painful, and can take up to 4 hours of time for the patient. The blue dyes have a variable success rate and can cause significant tissue staining. In the long term there may be permanent discoloration of the skin with some dyes [5]. The ICG is injected after induction of anesthesia, does not stain tissues. and travels rapidly to the nodal basin. There have been no reported complications from ICG at this dose. ICG is often visible through the skin prior to incision, unlike the blue dyes. Dissection of the nodal basin can occur with real time visualization of the nodes, leading to rapid and accurate identification of the sentinel node. This is particularly useful in situations such as a small deep node or a small node with a large radioactive signal.

Several recent studies have evaluated the use of ICG and real time lymphangiography for localizing sentinel nodes, mostly in patients with breast cancer [2, 3, 5–8, 15]. Additional studies have shown its effectiveness in melanoma and have even used ICG-radiocolloid conjugates for lymph node tracking [9–15]. ICG dye lymphangiography can reliably localize sentinel nodes in patients with melanoma and is well tolerated. There is a higher average number of sentinel nodes removed when using ICG versus tech99, which was true in our patient population as well. In the study by Fujisawa et al. an average of 2.18 sentinel nodes was removed with ICG versus 1.76 with tech99 [11]. Tagaya et al. found an average of 5.4 sentinel nodes per patient with ICG, although this group used a higher dose of ICG (5 mg) [2]. Hirche et al. found no difference in number of sentinel nodes, with 2 per patient in the ICG and tech99 groups [6]. In our study, the difference between 2.8 nodes with ICG and 2.6 nodes with tech99 is not felt to be clinically significant. In future studies the average number of nodes resected should continue to be monitored. The intensity of fluorescence may be able to serve as a cutoff for determining true sentinel nodes, just as the counts from the gamma probe do.

Our results with the tech99 were worse than expected from the published literature; however, this is likely due to the small sample size. During the time period of April 2010–January 2011 at our hospital a total of 92 patients with melanoma had 38 sentinel node biopsies, with successful localization of the sentinel node in 35 of the 38 patients or 92%. Two of the three unsuccessful localizations were captured in this patient population. The results with the methylene blue were also disappointing; however, Isosulfan blue dye is not available at our institution due to the cost.

There are certain limiting factors that will impact the utility of ICG dye lymphangiography as a primary method for the detection of SLNs. Hospitals would need to invest in a near-infrared ICG laser angiography device such as the SPY machine, which is available throughout the United States. This is the first study to our knowledge that has used the SPY system while others have used near infrared ICG laser angiography systems such as the FLARE and Photodynamic Eye systems [2, 14]. The retail cost of a SPY machine is $275,000, but the SPY machine has multiple other possible applications, increasing its utility. Another limitation may be time. After an hour, the ICG has diffused into the tissues, creating a high level of background signal. For single basin exploration this should be sufficient time, but if multiple basins need to be explored, it may become problematic. The two subjects in this series who localized to 2 nodal basins had an equivalent number of nodes per basin resected compared to the single basin patients. The optimal dose of ICG has not been fully explored and should be part of future studies with this method.

## 5. Conclusion 

Real time lymphangiography using ICG dye has the potential to simplify sentinel node localization for patients, without compromising patient safety or accuracy of staging. While the technology may require some additional development, this technique could potentially eliminate methylene blue, radioactive colloid, or both and result in reduced hospital trips, removal of radioactive material, and decreased tissue staining. Prospective studies with a larger number of patients are needed to better ascertain the full functionality of this technique.

## Figures and Tables

**Table 1:** Patient demographics including T stage, site of melanoma, the nodal basin(s), how many nodes were identified by each method, and the total number of nodes harvested. The number of malignant nodes is indicated, as well as which method identified these malignant nodes.

Patient	T stage	Primary site	Node basin	ICG+	MB+	Tech99+	Total nodes	Total imlignant nodes	Dye*
1	T2b	Anterior chest	Axilla	1	0	3	3	0	
2	T4b	Right calf	Groin	2	1	0	2	1	I, B
3	T3a	Left leg	Groin	1	1	1	1	1	I, T, B
4	T2a	Left temporal	Cervical	3	1	3	3	0	
5	T3b	Left chest	Axilla	0	0	0	1	0	
6	T3a	Scalp	Cervical	2	2	2	2	1	I, T, B
7	T4b	Right thumb	Axilla	2	1	2	2	0	
8	T3a	Left back	Axilla	3	1	2	3	0	
9	T2a	Left thigh	Groin	4	2	4	4	0	
10	T2a	Right back	Axilla	2	1	3	3	0	
11	T2a	Right flank	Groin	3	3	2	3	0	
12	T4a	Scalp	Bilat cerrical	4	3	3	4	0	
13	T2a	Left neck	Cervical	0	0	0	0	0	
14	T4b	Anterior chest	Bilat axilla	6	2	3	6	1	I
15	T3a	Left arm	Axilla	4	0	3	4	0	

Total				37	18	31	41	4	

Average # nodes per patient				2.8	1.6	2.6			

*Dye: I: ICG, B: MB, T: Tech99.

**Table 2:** Number of lymph nodes identified by each method.

	Total *N* (%)	Malignant *N* (%)
All nodes	40	4
ICG uptake	37 (93%)	4 (100%)
Tech99 uptake	31 (78%)	2 (50%)
MB uptake	18 (45%)	3 (75%)
Tech99 and MB combined uptake	40 (100%)	3 (75%)
ICG and Tech99 combined uptake	29 (73%)	2 (50%)
